# Determination of tricyclic antidepressants in human urine samples by the three-step sample pretreatment followed by HPLC-UV analysis: an efficient analytical method for further pharmacokinetic and forensic studies

**DOI:** 10.17179/excli2018-1613

**Published:** 2018-09-24

**Authors:** Ali Mohebbi, Mir Ali Farajzadeh, Saeid Yaripour, Mohammad Reza Afshar Mogaddam

**Affiliations:** 1 Department of Analytical Chemistry, Faculty of Chemistry, University of Tabriz, Tabriz, Iran; 2 Engineering Faculty, Near East University, 99138 Nicosia, North Cyprus, Mersin 10, Turkey; 3 Department of Pharmaceutical and Food Control, Faculty of Pharmacy, Urmia University of Medical Sciences, Urmia, Iran; 4 Department of Drug and Food Control, Faculty of Pharmacy, Tehran University of Medical Science, Tehran, Iran; 5 Food and Drug Safety Research Center, Tabriz University of Medical Sciences, Tabriz, Iran; 6 Pharmaceutical Analysis Research Center and Faculty of Pharmacy, Tabriz University of Medical Sciences, Tabriz, Iran

**Keywords:** tricyclic antidepressant, sample pretreatment, high performance liquid chromatography-ultraviolet detection, urine

## Abstract

In this work, an efficient sample pretreatment method has been developed by combining salt induced-homogenous liquid-liquid extraction, dispersive solid phase extraction, and dispersive liquid-liquid microextraction based on the solidification of floating organic droplet for the extraction of some widely used tricyclic antidepressant (TCA) drugs (nortriptyline, amitriptyline, desipramine, clomipramine, and imipramine) in human urine samples before their determination by high performance liquid chromatography-ultraviolet detection. In brief, the target analytes are first isolated from urine samples into acetonitrile (ACN) separated by adding a salt. Then the obtained ACN phase is treated with a mixture of appropriate sorbents to remove interferences. Afterward, the purified ACN is mixed with menthol as an extractant and rapidly injected into alkaline HPLC-grade water as a preconcentration step. Next, the obtained solution is placed in an ice bath and menthol collects on top of the solution after solidification. The solidified drop is then withdrawn and injected into separation system after dissolving in 10 µL ACN. Under the optimum experimental conditions, extraction recoveries and enrichment factors of the selected drugs ranged from 69-84 % and 345-420, respectively. The limits of detection and quantification were obtained at the ranges of 0.22-0.31, and 0.71-1.1 µg L^-1^, respectively. The relative standard deviations of the proposed method were ≤ 6 % for intra- (n=6) and inter-day (n=4) precisions at a concentration of 10 µg L^-1^ (each drug). Finally, the suggested approach was applied to determine of TCA drugs in different patients' urine samples. The method could be applied in further TCAs pharmacokinetic and forensic studies.

## Introduction

Millions of people all over the world suffer from a common mental disorder named depression. This disorder causes patients to experience serious problems such as depressed mood, feelings of guilt, disturbed sleep, low energy, and loss of interest or pleasure. In the 1950s, antidepressants were developed which could elevate patients' mood. Antidepressants are commonly classified in two main groups: the monoamine oxidase inhibitors and TCAs (Parfitt and Martindale, 2002[11]; Micó et al., 2006[9]). TCAs are one of the oldest classes of antidepressants and impede the reuptake of serotonin and norepinephrine (Furlanut et al., 1993[5]). The drug concentrations in biological fluids play a critical role in pharmacotherapies. This role is much more important in the case of TCAs, due to their narrow therapeutic range. So, improving of the sensitivity and detection limit of the analytical method is necessary to determine the concentrations of TCAs in biological fluids (plasma, serum or urine) in order to obtain the best therapeutic concentration for effective control of pharmacotherapy and drug poisoning. 

Chromatographic techniques such as gas (Gupta et al., 1983[6]; Yazdi et al., 2008[20]) and liquid chromatography (Woźniakiewicz et al., 2008[17]; Zheng et al., 2010[23]) are suitable for these purposes. However, because of the complicated matrices of the biological samples and low concentration of the selected drugs in them, a selective preparation approach is required before their determination by chromatographic techniques. In the past years, solid phase extraction (SPE) and liquid-liquid extraction were applied for the extraction and preconcentration of the target analytes from different matrices. However, these methods require high volumes of sample and organic solvents and are environmentally unfriendly and time-consuming (Yazdi and Amiri, 2010[19]; Poole, 2003[12]). Homogeneous liquid-liquid extraction (HLLE) is an extraction procedure in which analytes existing in a homogeneous solution are extracted into a water-immiscible solvent by adding a phase separation agent. Temperature adjusting, salt addition or pH adjustment are mostly used as the phase separation agents (Murata et al., 1972[10]; Jain et al., 2015[7]; Zhao et al., 2009[22]; Tavakoli et al., 2008[15]). The main disadvantage of HLLE-based methods is that they are not selective, which limits their application to simple, aqueous samples. In order to overcome this problem, a clean-up step is often required for the removal of interfering substances such as organic acids, pigments, and sugars. Dispersive solid phase extraction (DSPE) is an efficient clean-up method which is based on SPE. But unlike SPE, in this method the sorbent is directly added to the extract without conditioning and pretreatment (Wu et al., 2011[18]; Díez et al., 2006[3]). However, low enrichment factors (EFs) are achievable by performing HLLE. In recent years, different preconcentration microextraction methods including solid phase microextraction (Zhang et al., 1994[21]) and liquid-phase microextraction (LPME) (Psillakis and Kalogerakis, 2003[13]) have been introduced. In 2006, dispersive liquid-liquid microextraction (DLLME) was introduced as a novel mode of LPME (Rezaee et al., 2006[14]). In DLLME, an appropriate mixture of an extraction and a disperser solvent is rapidly injected into an aqueous solution containing analyte. As a result, tiny droplets of the extractant are formed and the analytes are extracted into these drops. Although DLLME eliminates some of the problems of the traditional methods, toxic organic solvents like chloroform, carbon tetrachloride, chlorobenzene, and etc. are required for this method, though much less than the traditional extraction methods. Later, a DLLME method based on the solidification of a floating organic drop (DLLME-SFO) was established by means of lighter than water solvents (Leong and Huang et al., 2008[8]). DLLME-SFO is similar to conventional DLLME, except that after centrifugation, floating phase on top of aqueous solution is solidified with the help of an ice bath. Solvents such as 1-dodecanol, and 1-undecanol which possess melting points near to room temperature are commonly used in this method (Dai et al., 2010[2]).

The purpose of the presented work is to introduce feasible, efficient, and green sample pretreatment approach based on the combination of salt induced-HLLE (SI-HLLE) with DSPE as a clean-up step, and DLLME-SFO using menthol as a safe solvent in order to extract of some TCAs from urine samples before their determination by high performance liquid chromatography-ultraviolet detector (HPLC-UV). The proposed method simultaneously possesses the advantages of SI-HLLE, DSPE, and DLLME-SFO. The use of menthol as an easily-accessible and green extractant in place of aromatic and halogenated solvents makes the approach eco-friendly. The effect of different parameters on the extraction efficiency of the suggested method were thoroughly studied and optimized.

## Materials and Methods

### Reagents and solutions

Nortriptyline and amitriptyline were purchased from Daroupakhsh Company (Tehran, Iran). Desipramine was a gift from Exir Pharmaceutical Company (Boroujerd, Iran). Clomipramine and imipramine were obtained from Soha Pharmaceutical Company (Karaj, Iran). Sodium sulfate, sodium chloride, potassium chloride, ammonia (25 %), hydrochloric acid (37 %), acetone, dimethyl formamide (DMF), sodium hydroxide, and ammonium acetate were supplied from Merck (Darmstadt, Germany). Menthol with a purity of 98 % was also purchased from Merck. Primary-secondary amine (PSA), graphitized carbon black (GCB), and octadecylsilane (C_18_) sorbents were purchased from Agilent Technologies Company (Santa Clara, CA, USA). Methanol, acetonitrile (ACN), and HPLC-grade water were supplied from Chemlab (Zedelgem, Belgium). A mixture stock solution of drugs was prepared in methanol with a concentration of 50 mg L^-1^ (each drug) and working standard solutions were prepared daily by diluting the stock solution with HPLC-grade water. 

### Apparatus

Hewlett Packard 1090-II HPLC system (Palo Alto, CA, USA) equipped with a fixed wavelength UV-vis detector was used as the analytical instrument. Chromatographic separation of the analytes was carried out on an RP-C_18_ column obtained from Shimadzu (Kyoto, Japan) (15 cm × 4.6 mm i.d. with particles size of 5 µm). The mobile phase was a mixture of ammonium acetate (0.05 M, adjusted at pH 5.5) -ACN (55:45, *v/v*) at a flow rate of 0.5 mL min^-1^ using an isocratic elution. Monitoring of the drugs was done at 230 nm. All injections were performed manually using a 10-µL sample loop. The HPLC system was controlled using ChemStation software. A Metrohm pH meter (model 654, Herisau, Switzerland), Hettich centrifuge (model D-7200, Kirchlengern, Germany), and an L46 vortex (Labinco, Breda, Netherlands) were used in pH adjustment, accelerating phase separation, and vortexing the samples, respectively.

### Samples

Blank urine sample was obtained from a healthy volunteer who had not taken any drugs for at least one month. Three urine samples were obtained from three depressed female patients being treated with amitriptyline (25 mg, twice in a day), clomipramine (50 mg, per day), and nortriptyline (25 mg, twice in a day), respectively. In addition, two other distinct blood and urine samples were obtained from two depressed male patients being treated with desipramine (25 mg, twice a day), and imipramine (25 mg, twice a day), respectively. The volunteers were well informed of the study and signed written consent forms. All of the samples were collected within 24 h from first oral administration. All samples were collected in polypropylene vessels and stored at -20 °C until use. The urine samples were adjusted at pH 10.0 with the help of an ammoniacal buffer (0.5 M) and then subjected to the proposed method.

### Extraction procedure

#### SI-HLLE

Five mL ammoniacal buffer (0.5 M, pH=10) spiked with the investigated drugs (20 µgL^−1^, each drug) or the urine sample (see Samples) was transferred into a 10-mL glass test tube. Afterward, 2.0 mL ACN was added to it and a homogenous solution was obtained. Then 1.75 g sodium sulfate (35 %, *w/v*) was added to the homogenous solution and vortexed until it was dissolved completely. By this action, phase separation occurred and fine droplets of ACN containing the extracted analytes were formed and collected on the surface of aqueous phase (1.1 ± 0.05 mL) after centrifuging for 5 min at 5000 rpm. 

#### DSPE

One mL of the ACN obtained from the previous step was removed using a 1-mL glass syringe and transferred into a 10-mL glass test tube containing 50 mg PSA, 25 mg GCB, and 50 mg C_18_. The solution was vortexed for 2 min and centrifuged at 5000 rpm for 5 min. Then the supernatant was removed and used in DLLME-SFO.

#### DLLME-SFO

**Figure 1:**
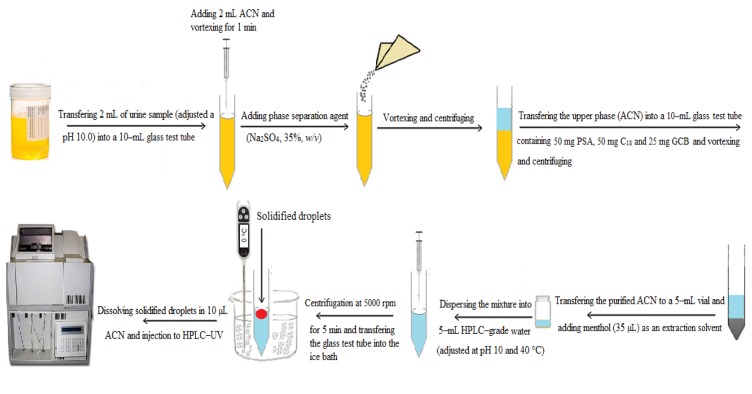
Schematic procedure of the developed method

The ACN phase obtained from the DSPE step was mixed with 35 µL menthol (extraction solvent) and rapidly injected into 5 mL ammoniacal buffer (adjusted at pH=10.0 and temperature of 40 °C) placed in a 10-mL glass test tube. By this action, fine droplets of menthol were formed and the target analytes were extracted into them. The solution was centrifuged at 5000 rpm for 5 min. Afterward, the solution was placed in an ice bath and the menthol was collected on top of the solution as a solid drop. This drop was transferred into a vial with a spatula and dissolved in 10 µL ACN. At last, the solution was injected into determination system. The developed method procedure is shown in Figure 1(Fig. 1).

## Results and Discussion

### Optimization of SI-HLLE step

#### Selection of extraction/disperser solvent type and volume

In the present work, the extraction solvent used in SI-HLLE step acts as a disperser in the following preconcentration step (DLLME-SFO). Considering this fact, the selected solvent should meet the following criteria: (1) ability of a two-phase system formation after salt addition, (2) high extraction efficiency for the target drugs, (3) miscibility with extractant used in DLLME*-*SFO and aqueous phase, (4) density lower than water, and (5) low toxicity. Considering these properties, four organic solvents including ACN, methanol, acetone, and DMF (2.0 mL each of them) were selected and tested in this step. The obtained results indicated that only by using ACN is a two-phase system formed after the addition of sodium sulfate (30 %, *w/v*). Consequently, ACN was chosen in this step. 

**Figure 2:**
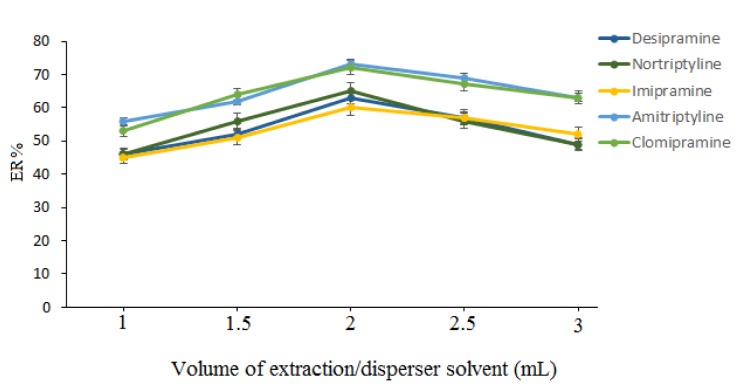
Selection of extraction/disperser solvent. Extraction conditions: aqueous sample volume, 5 mL HPLC–grade water spiked with 20 µg L^–1^ of each analyte; extraction/disperser solvent, ACN; phase separation agent, sodium sulfate (30 %, w/v); aqueous phase pH value, 10.0; vortex time, 1 min; sorbent (amount), PSA (50 mg); vortex time, 1 min; aqueous phase in DLLME–SFO, 5 mL HPLC–grade water; aqueous phase temperature (50 °C); aqueous phase pH, 10.0; extractant (volume), menthol (40 µL); centrifugation rate and time 5000 rpm and 5 min; and aqueous phase pH value, 10.0.

ACN volume is another parameter that can influence the efficiency of the proposed method. To investigate this parameter, different volumes (1-3 mL with 0.5-mL intervals) of ACN were evaluated. It is obvious that the initial volume of ACN can affect the volume of the separated phase after adding phase separation agent. According to the obtained results, in the cases of 1.0, 1.5, 2.0, 2.5, and 3.0 mL, the volume of the separated phase was 0.2, 0.5, 0.9, 1.2, and 1.5 mL, respectively. In these experiments, 1 mL of the separated phase was removed and used in the next steps, except in the cases that lower than 2.5 mL of ACN was used and the volumes of the collected phases were lower than 1 mL. In those cases, all of the collected phase was removed and mixed with appropriate volumes of pure ACN in order to reach the final volume of 1 mL and was used in other cases. The experimental results in Figure 2(Fig. 2) indicate that the extraction recoveries (ERs) of the selected drugs increased by increasing the volume of the ACN from 1.0-2.0 mL and then slightly decreased between 2.0 and 3.0 mL. Subsequently, 2.0 mL ACN was used in the next experiments.

#### Selection of type and concentration of phase separation agent

In SI-HLLE method, the type and concentration of salt play key roles. In most cases, salt addition decreases the solubility of the analytes in the sample solution and simultaneously increases distribution of the analytes into the organic phase, which leads to an increase in the extraction efficiency of the target analytes. In addition, in the proposed method the obtained homogeneous solution is broken by dissolving an appropriate amount of a salt as the phase separation agent. To evaluate this factor, three diverse salts including sodium sulfate, potassium chloride, and sodium chloride (30 %, *w/v, *of each salt) were studied. The obtained results did not show a significant variation (*p *values > 0.05) in the extraction efficiency of the target analytes using the mentioned salts. But, due to the higher solubility of sodium sulfate in the aqueous phase compared to the other tested salts, it was selected in this step.

**Figure 3:**
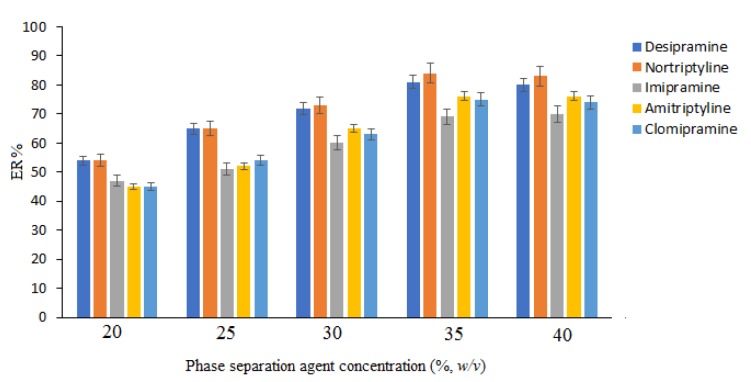
Study of phase separation agent concentration. Extraction conditions: are the same as those used in Figure 2, except that 2 mL ACN was used as an extraction/disperser solvent.

In order to investigate the effect of salt concentration, the concentration of sodium sulfate was changed in the range of 20 to 40 %, *w/v*. It is obvious that the concentration of sodium sulfate can affect the volume of the separated phase. According to the obtained results, in the cases of 20, 25, 30, 35, and 40 ( %, *w/v*), the volume of the separated phase was 0.2, 0.5, 0.9, 1.1, and 1.4 mL, respectively. In all cases, 1.0 mL of that phase was used in the subsequent steps, except in the cases of concentrations lower than 35 %, *w/v*, in which the volumes of the collected phases were lower than 1 mL. In these cases, all of the collected phase was removed and mixed with appropriate volumes of pure ACN in order to reach the final volume of 1 mL and was used in the other cases. As can be seen in Figure 3(Fig. 3), extraction efficiency of the studied drugs increases by enhancing the sodium sulfate concentration up to 35 % (*w/v*) and afterward remain constant considering *p* values > 0.05. Hence, 35 % (*w/v*, sodium sulfate) was selected in this step. It is noted that in concentrations less than 20 % (*w/v,* sodium sulfate), phase isolation was not seen.

### Study of pH

The efficiency of the HLLE based methods in extracting the analytes which are acidic or basic, may be altered with the change in pH of the aqueous solution. In this study, pH was studied by varying the pH of the aqueous solution from 3 to 12 adjusted by 1 M HCl or NaOH solution.

Regarding the results, the extraction efficiency of the selected drugs improved by increasing the aqueous phase pH from 3 to 10 and afterward remained constant considering *p* values > 0.05. This can be attributed to the fact that the selected drugs are much easily extracted in their neutral forms compared to ionic forms. Regarding the pK_a _values of the selected drugs (Alves et al., 2006[1]), at pHs lower than pK_a_ the analytes are in their ionic forms due to protonation of amine groups in the sample solution and have less tendency to be extracted. Therefore, the pH of the samples was adjusted at 10.0 in the next experiments. To simplify the pH adjustment, an ammoniacal buffer (0.5 M, pH= 10.0) was used.

### Optimization of DSPE step

DSPE as a clean-up method may decrease the matrix interferences of urine samples before performing the preconcentration step. The efficiency of DSPE in removing matrix interferences depends on the type and amount of the selected sorbents which adsorb interferences and allow the compounds of interest to maintain in organic phase. In the proposed approach, 1 mL of the ACN obtained from SI-HLLE step was transferred into a 10-mL glass test tube containing the sorbents. The single, dual, and triple systems of C_18_, PSA, and GCB were tested as the sorbent for the selected analytes. A single system of 50 mg C_18_, 50 mg PSA, or 25 mg GCB did not give sufficient clean chromatograms to the urine samples. To improve the clean-up effect, the dual and triple sorbent systems, including C_18_-GCB, PSA-GCB, C_18_-PSA, and C_18_-PSA-GCB were evaluated. The obtained results indicated that the mixture of C_18_-PSA-GCB was the best among the others and removed interfering materials efficiently and gave very clean chromatograms top the urine samples. So, a triple system of the sorbents including 50 mg C_18_, 50 mg PSA, and 25 mg GCB was selected for the subsequent experiments.

### Optimization of DLLME-SFO step

#### Selection of extraction solvent and its volume

The extraction solvent has a main effect on the extraction capability of analytes in DLLME-SFO. In this method, extraction solvent should satisfy the following criteria: lower density than water, low toxicity, good chromatographic behavior, low solubility in water, and melting point near to room temperature. Based on these necessities, menthol was evaluated as an extractant in this step. Menthol (C_10_H_20_O), is a green and safe (LD_50_ oral/rat 3300 mg kg^-1^) organic compound with a density of 0.9 g cm^-3 ^which helps it float on the surface of water. It has a melting point of 36-38 °C causing its easy solidification. Also, it is slightly soluble in water. Therefore, menthol was used as an extractant in the proposed approach.

Extractant (menthol) volume is another main parameter that can influence the extraction capability and LODs of the method. To study the effect of this parameter, different volumes of menthol were tested in the range of 35-50 μL. According to the obtained results, ERs of the analytes remained constant as menthol volume was enhanced from 35 to 50 μL. Considering these facts, 35 μL was selected in this step.

### Optimization of salt effect

Salt addition is another critical parameter that should be optimized. This phenomenon can induce a salting out effect in which the analyte solubility decreases and as a result, the extraction capability of the target analytes improves. In the present work, sodium sulfate was selected to adjust the ionic strength of HPLC-grade water due to its higher solubility in comparison with other salts like potassium chloride, and sodium chloride. To study this parameter, various concentrations of sodium sulfate (0-10 %, *w/v*) were tested. According to the results, ERs of the analytes decreased with the increase in sodium sulfate concentration. So, subsequent experiments were conducted without using sodium sulfate in this step.

### Optimization of aqueous phase temperature

In the suggested approach, diffusion and distribution coefficients of the analytes can be varied by changing the aqueous solution temperature. This phenomenon can help with better dispersion of menthol into the aqueous solution. Therefore, it can affect the extraction capability of the selected analytes, so it should be optimized. To study this parameter, various experiments were performed in the temperature range of 30 to 50 °C. It should be noted that at temperatures less than 30 °C, the method became useless because the solution had no turbidity. According to the results, the extraction capability of the selected analytes increased up to 40 °C and, afterward, remained constant. So, 40 °C was used in the subsequent tests.

### Evaluation of other parameters

Centrifugation is a commonly used procedure to obtain fast isolation of extractant droplets from aqueous solution. The effects of centrifugation time and rate were investigated in the ranges of 2-6 min and 3000-6000 rpm, respectively, three times in this study. According to the obtained results, 5000 rpm and 5 min were selected as the centrifugation rate and time, respectively in all steps.

In the proposed method, vortex agitation was used to improve the contact area between sorbents and ACN. It was evaluated in the range of 0.5 to 3 min. Considering the results, 2.0 min was selected in this step.

### Method validation

The suggested method was validated by evaluating numerous parameters such as limit of detection (LOD), limit of quantification (LOQ), accuracy, linearity, selectivity, intra- and inter-day precisions, stability, and EFs and ERs using international guidelines and protocols (U.S. Food and Drug Administration, 2017[16]; European Medicines Agency, 2017[4]).

### Calibration curves and linearity

To obtain the linearity of the developed approach, matrix-matched calibration curves were plotted using peak area *versus* analytes concentrations of the urine sample. The LOD and LOQ values were examined on the basis of the signal-to-noise ratios (S/N) of 3 and 10, respectively. Lower limit of quantification (LLOQ) was informed as the lowest concentration on the calibration curve that could be determined with the accuracy of 80-120 % and the precision of relative standard deviation (RSD) ≤ 20 %. Good linearities were obtained with coefficient of determination ≥ 0.996. The LODs, LOQs, and LLOQs values are low, which indicate that the suggested method can be used to determine the target analytes in urine. The attained results are indicated in Table 1(Tab. 1).

### Accuracy and precision

Accuracy and precision are defined as the measurements of the systematic and random errors, respectively. Precision stated as RSD % was investigated by applying suggested approach on six quality control (QC) samples (for intra-day) and four QC samples (for inter-day) at the concentration of 10 μg L^-1^ of each drug and ranged from 2-4 % and 3-6 %, respectively. Accuracy was determined by added-found method using five replicate determinations at 10 μg L^-1^ level of each analyte, and the obtained deviations were less than 6 %. 

### Selectivity

Evaluating the effects of the compounds that can be available in urine to show the capability of the method in measuring analytes in the presence of these components is defined as selectivity. The interference of drugs that can potentially be available in urine was studied to evaluate matrix exogenous substances by spiking the blank urine with 10 mg L^-1 ^of each drug. The drugs tested in the selectivity assay were antiepileptic drugs (carbamazepine, and valproic acid), anti-inflammatory drugs (ibuprofen, acetaminophen, sodium diclofenac, and naproxen), antiarrhythmic drugs (propranolol, metoprolol, verapamil, and carvedilol), and antidepressants (trimipramine, doxepine, and paroxetine). The obtained results confirmed that there is no endogenous interference in the retention times related to the evaluated analytes. Responses of the analytes at the LLOQ concentrations were compared with the responses of the samples spiked with mentioned drugs. No interference from the mentioned drugs was observed for the studied analytes. These results indicate that the proposed method is selective for the analysis of analytes in urine. 

### Stability

To evaluate the stability of the selected drugs in the urine samples, the blank urine was spiked with drugs at the concentrations of 50 and 100 μg L^-1^ (each drug, n=3). In this work, short- and long-term stabilities were determined by analyzing the samples kept at room temperature (24 °C) for 12 hours and stored at -20 °C for 5 days, respectively. The freeze-thaw stability of the analytes was also determined after three freeze and thaw (-20 to 24 °C) cycles. The obtained results were compared with those of freshly prepared samples and were expressed in RSD %. The obtained RSD % were less than 7 %, which indicates good stability of the selected drugs in urine under the studied conditions.

### Calculation of EFs and ERs

EF is stated as the ratio between the analyte concentration in the collected organic phase (C_coll_) and the initial concentration of the analyte (C_0_) in aqueous solution.


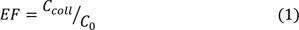


ER is stated as the percentage of the total analyte amount (n_0_) that is extracted into collected phase (n_coll_):


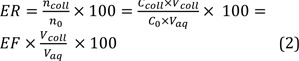


In this equation V_coll_ and V_aq_ are volumes of the collected phase and sample solution, respectively. High EFs (345-420) and good ERs (69-84 %) were obtained in this study (Table 1(Tab. 1)).

### Study of matrix effect 

To evaluate the matrix effect in the urine sample, the added-found method was used. The blank urine sample was spiked with the selected analytes at three concentration levels (10, 50, and 100 µg L^−1 ^of each analyte) and the suggested method was applied to it for triplicate. In this step, the results obtained for the evaluated drugs in the urine sample are compared with those attained for HPLC-grade water spiked at same concentrations and expressed as relative recoveries (Table 2(Tab. 2)). Regarding the results, the suggested method indicates good recoveries in the urine sample ranging from 84 to 97 % and is relevant and applicable for the determination of target drugs in urine. 

### Real samples analysis

**Figure 4:**
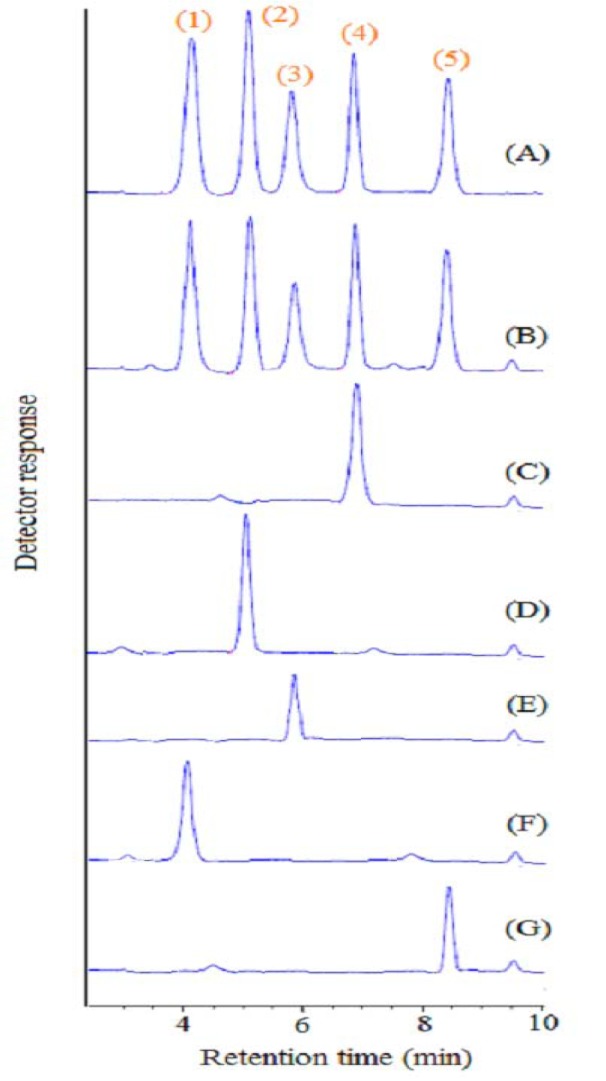
Typical HPLC-DAD chromatograms of: (A) standard solution of the selected analytes at a concentration of 20 mg L^-1^ of each analyte (direct injection), (B) blank urine sample spiked with 50 µg L^−1^ of each analyte, (C) urine sample of a female patient treated with amitriptyline, (D) urine sample of a female patient treated with nortriptyline, (E) urine sample of a male patient treated with desipramine, (F) urine sample of a male patient treated with imipramine, and (G) urine sample of a female patient treated with clomipramine. Detection wavelength was 230 nm. For more chromatographic conditions see the experimental section. Peaks identification: 1) desipramine, 2) nortriptyline, 3) imipramine, 4) amitriptyline, and 5) clomipramine.

To evaluate the applicability of the introduced approach, the urine samples of three depressed female patients who are being treated with amitriptyline tablets (25 mg, twice a day), nortriptyline (25 mg, twice a day), and clomipramine (50 mg, once a day), respectively, were analyzed. In addition, the suggested approach was applied to the analysis of urine samples of two depressed male patients treated with desipramine (25 mg, twice a day) and imipramine (25 mg, twice a day), respectively. Figure 4(Fig. 4) shows HPLC-UV chromatograms of these samples after applying the suggested approach as well as the blank urine sample and direct injection of a standard solution of analytes (20 mg L^-1^ of each analyte) in methanol. After three determinations of each sample using standard addition method, the detected concentrations of amitriptyline, nortriptyline, desipramine, clomipramine, and imipramine in the evaluated urine samples were 35 ± 2, 38 ± 4, 29 ± 2, 39 ± 3, and 40 ± 4 µg L^-1^, respectively. 

## Conclusion

In this study, a green and efficient approach (SI-HLLE-DSPE-DLLME-SFO) was developed for the extraction of some TCAs in urine samples prior to their determination by HPLC-UV. Throughout the proposed method, no toxic organic solvent was used. Small volumes of non-toxic solvents like ACN and menthol were consumed and helped decrease the risk for human health and environment. The validation results indicated high sensitivity, accuracy, and precision for the suggested approach. The proposed procedure could be applied as an efficient analytical method in further TCAs pharmacokinetic and forensic studies.

## Acknowledgement

Authors are grateful to Research Council of the Tabriz University of Medical Sciences for financial support.

## Conflict of interest

The authors declare that they have no conflict of interest.

## Figures and Tables

**Table 1:**
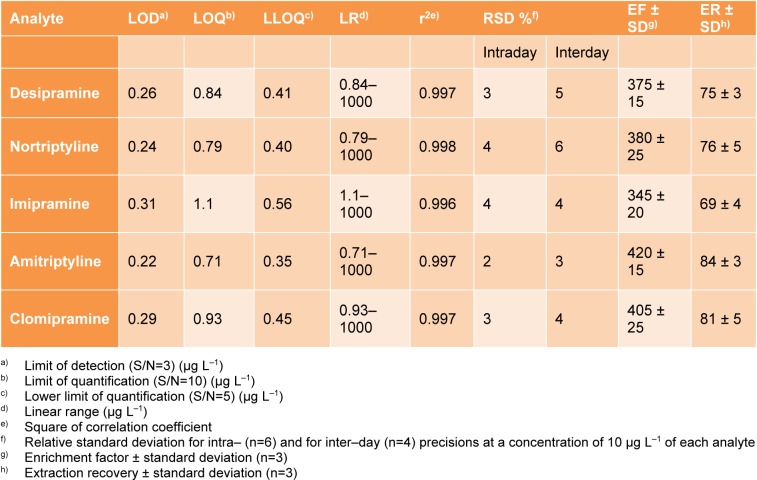
Quantitative features of the developed method for the selected tricyclic antidepressant drugs

**Table 2:**
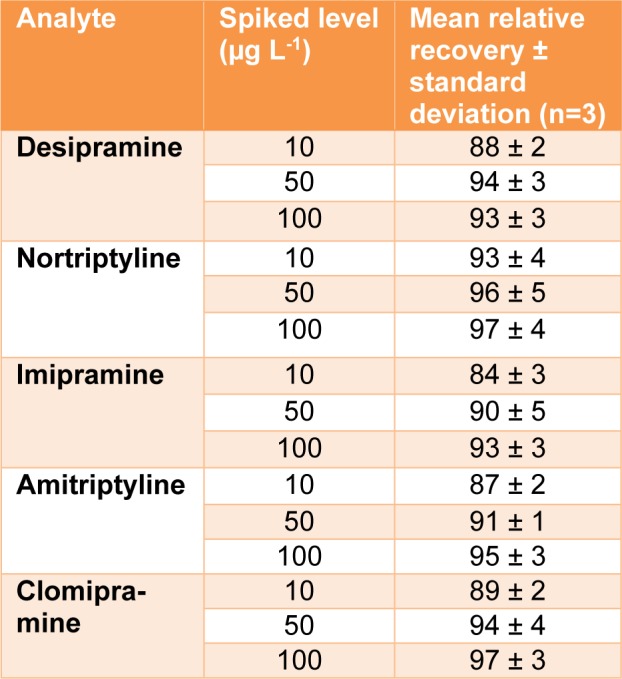
Study of matrix effect by the proposed method in the blank urine sample spiked at different concentration levels.
